# Moderate Wine Consumption, Defined by the Mediterranean Diet, Is Associated With Delayed Biological Aging in Men From the Moli-sani Study

**DOI:** 10.3389/ijph.2026.1609410

**Published:** 2026-03-16

**Authors:** Simona Esposito, Augusto Di Castelnuovo, Simona Costanzo, Alessandro Gialluisi, Antonietta Pepe, Emilia Ruggiero, Amalia De Curtis, Sara Magnacca, Mariarosaria Persichillo, Francesc Casanovas-Garriga, Chiara Cerletti, Maria Benedetta Donati, Giovanni de Gaetano, Licia Iacoviello, Marialaura Bonaccio

**Affiliations:** 1 Research Unit of Epidemiology and Prevention, IRCCS Neuromed, Pozzilli, Isernia, Italy; 2 Department of Medicine and Surgery, LUM University, Bari, Italy; 3 Department of Medicine and Surgery, Research Center in Epidemiology and Preventive Medicine (EPIMED), University of Insubria, Varese, Italy; 4 Department of Internal Medicine, Hospital Clínic de Barcelona, University of Barcelona (UB), Barcelona, Spain; 5 Institut de Recerca en Nutrició i Seguretat Alimentaria (INSA-UB), University of Barcelona (UB), Barcelona, Spain; 6 Institut d’Investigacions Biomèdiques August Pi i Sunyer (IDIBAPS), Barcelona, Spain

**Keywords:** biological aging, mediterranean diet, neurological decline, prevention, wine consumption

## Abstract

**Objectives:**

To investigate the association between wine consumption and biological aging in the Moli-sani Study.

**Methods:**

Dietary data were assessed using a 188-item FFQ. Participants (n = 22,495) were classified as abstainers, former drinkers, moderate drinkers according to national guidelines (≤250 mL/d men; ≤125 mL/d women) or Mediterranean Diet (MD) (125–500 mL/d men; 62.5–250 mL/d women), and heavy drinkers (>500 mL/d men; >250 mL/d women). Biological age (BA) was estimated with a deep neural network using 36 circulating biomarkers, and Δage (BA–chronological age) served as an index of biological aging.

**Results:**

In men, wine consumption, at doses defined moderate by a current MD Score, was associated with slower biological aging (Δage β = −0.39; 95%CI: −0.78, −0.01 vs. abstainers). Dose–response analyses showed a J-shaped curve, with the slowest Δage at ∼170 mL/d (Δage = −0.34 years; 95%CI: −0.66, −0.03). Overall ethanol intake, including all alcoholic beverages consumed, was neutral at moderate levels and associated with faster biological aging at higher doses.

**Conclusion:**

Moderate wine consumption, but not overall ethanol intake, may contribute to slower biological aging in men.

## Introduction

Aging is a natural process characterized by a progressive decline in physiological functions, leading to multiple alterations across the body and varying between individuals [1].

To account for these differences, the concept of biological age (BA) has been proposed as a useful measure of health status [2]. Evidence suggests that BA, rather than chronological age (CA), better predicts disease risk and mortality [3]. BA can be estimated using different approaches, including DNA methylation, telomere length, blood-based measures, or composite indices derived from clinical biomarkers [4–6]. The difference between BA and CA, termed Δage, indicates whether an individual is aging faster or slower than expected; a positive Δage reflects accelerated aging, whereas a negative Δage suggests slower, potentially healthier aging [3].

Lifestyle and environmental factors, such as smoking [7] and obesity [8], are linked to faster BA, while adherence to healthy dietary patterns, including the Mediterranean Diet (MD), and consumption of polyphenol-rich foods have been associated with slower biological aging [9, 10].

Among lifestyle factors that may modulate BA, moderate wine consumption, particularly in the context of a traditional MD, represents a potentially beneficial behavior, due to its reported cardiovascular benefits [3, 11].

Although excessive alcohol consumption is a well-established risk factor for various chronic diseases [12], regular and moderate wine intake during meals is a hallmark of the traditional MD [13, 14] and has been associated with improved cardiovascular outcomes and longevity [15, 16]. An inverse association of biological aging with alcohol consumption has already been reported in previous findings from the same Moli-sani cohort where moderate alcohol consumption showed a deceleration of biological aging [3].

While the concept of “moderate consumption” varies across contexts, the traditional MD typically involves higher, but still moderate, ethanol intake, mainly in the form of red wine during main meals, compared to many national drinking guidelines in Europe [13]. Yet, some other organizations, as the World Health Organization, have stated that no level of alcohol consumption can be considered safe [17]. The potential mechanisms by which moderate wine intake may confer health benefits, particularly improved cardiovascular outcomes, include the anti-inflammatory and antioxidant effects of bioactive compounds such as resveratrol [18], favorable modulation of lipid profiles, particularly HDL cholesterol [19], enhanced endothelial function [20], reduced platelet aggregation [21], and improved insulin sensitivity [22]. These effects may be reflected in circulating biomarkers related to inflammation, lipid metabolism, and vascular function, processes whose collective patterns are synthetically captured by biological age measures [23], potentially representing pathways through which moderate wine consumption contributes to longevity and reduced disease risk.

To date, no study has directly investigated the association between wine consumption and biological aging based on circulating biomarkers, whereas previous research has primarily focused on ethanol intake in the context of at-risk or heavy drinking patterns [24–26].

To address this knowledge gap, we investigated the relationship between patterns of wine consumption within the context of a traditional MD and biomarkers-based biological aging in a well-established population cohort in Southern Italy.

## Methods

### Study Population

We analyzed data from the Moli-sani Study, a large population-based cohort study conceived to investigate genetic and environmental risk factors in the onset of chronic diseases. Between 2005 and 2010, a total of 24,325 individuals (aged ≥35 years, 52.0% females) were randomly recruited from city-hall registries of the Molise region, in the Central-Southern part of Italy. Additional details of the study design are available elsewhere [27]. The Moli-sani Study complies with the Declaration of Helsinki and was granted the approval of the Ethics Committee of the Catholic University in Rome, Italy.

### Blood Sample Collection, Computation of Biological Age and Selection of the Analytic Sample

Blood samples were collected at baseline in participants who had fasted overnight and had refrained from smoking for at least 6 h.

To compute BA, we used a supervised machine learning algorithm called Deep Neural Network (DNN) built in R (https://www.r-project.org/) through the Keras package (https://cran.r-project.org/web/packages/keras/index.html). We first carried out a quality control (QC) and elaboration of available data (N = 24,325), as briefly described below. Unreliable blood markers levels - i.e., leukocyte counts whose fractions summed <99% or >101% - were set to missing, and missing values were imputed through either a k-nearest neighbour (kNN) algorithm or a minimum/maximum imputation, as appropriate. Composite variables like total cholesterol, plateletcrit, haematocrit and mean corpuscular hemoglobin were removed to avoid collinearity, as were participants reporting non-Italian ancestry and/or non-faster status at the time of blood draw. This left 23,858 participants passing QC, with 36 circulating markers available for analysis, including lipid biomarkers (triglycerides, high and low density lipoprotein-cholesterol, lipoprotein A and apolipoprotein A1 and B); markers of glucose metabolism (glucose, C-peptide and insulin; liver enzymes, aspartate transaminase and alanine aminotransferase); cardiac and vascular markers (NT-proB-type Natriuretic Peptide and high-sensitivity cardiac troponin I); other hormones (testosterone and vitamin D); haemostasis markers such as D-Dimer; renal markers (uric acid, albumin, creatinine, cystatin-C); inflammation markers like high sensitivity C-reactive protein; common haemochrome markers (red blood cell count and distribution width, haematocrit, haemoglobin levels, mean corpuscular volume, mean corpuscular haemoglobin concentration, total white blood cells, lymphocytes, monocytes, granulocytes, neutrophils, basophils and eosinophils, platelet count, mean platelet volume and platelet distribution width). The 36 biomarkers used reflect all major physiological systems of the body, providing a comprehensive overview of multiple biological districts and systemic functions, namely, glucose homeostasis, sarcopenia, heart, renal and liver functionality [28]. These variables underwent min-max normalization before analysis and were used as input features of the DNN for the prediction of BA, along with recruiting center and sex of each participant, using CA as a label. In the original work where the DNN algorithm was deployed [23], we split the available QC dataset (N = 23,858) into a random training set – a portion of the cohort (80%, N = 19,086) where the DNN algorithm had known labels (CA) and was trained over 1,000 epochs to minimize Mean Squared Error between CA and BA (loss function) – and a test set - remaining 20% of the cohort, N = 4,772, where the label is initially unknown and is used to validate the deployed algorithm. In the test set, the BA measure resulting from the optimized DNN showed a Mean Absolute Error (MAE) of 6.00 years compared to CA, a Pearson’s correlation (r) of 0.76 and a Nagelke’s coefficient of determination (R2) of 0.57. Details on QC, DNN architecture and features used are reported in [23].

For the purpose of the present analysis, we used BA for the totality of samples passing QC (N = 23,858). We then excluded individuals with missing data on diet (n = 97), those reporting implausible energy intakes (<800 or >4,000 kcal/d in males, and <500 or >3,500 kcal/d in females; n = 660), or with medical and dietary questionnaires judged as unreliable by the interviewers (n = 233 and n = 939, respectively) [9, 29, 30]. The final sample size available for analysis was 22,495 participants (Supplementary Figure S1).

### Dietary Assessment and Definition of Patterns of Wine Consumption

Food intake during the year before enrolment was assessed by the EPIC food frequency questionnaire (FFQ) validated and adapted to the Italian population. The FFQ contains 14 sections with 248 questions concerning 188 different food items [31]. Using a specifically designed software, frequencies and quantities of each food were linked to Italian Food Tables [32] to obtain estimates of daily intake of macro- and micro-nutrients plus energy.

The average volume of alcohol consumed during the same period was assessed through the same validated Italian EPIC FFQ [31], supplemented with specific additional questions [33]. For the calculation of ethanol intake, it was assumed that 12 g of ethanol correspond to 120 mL of wine, 330 mL of beer, or 40 mL of spirits.

Alcohol units were defined according to the Italian dietary guidelines issued by the Council for Agricultural Research and Economics (CREA), which defines one alcoholic unit as equivalent to 125 mL of wine, containing approximately 12 g of ethanol [34].

Based on the reported amounts of wine consumed, participants were then classified into five categories: a) abstainers; b) former drinkers, i.e., participants who reported current abstention but had ever consumed alcohol; c) moderate drinkers according to national drinking guidelines: in countries such as Germany, Greece, and Spain, these corresponded to allowances of up to 250 mL/d of wine for men, and 125 mL/d for women [17]; d) moderate drinkers according to the definition of a traditional MD, i.e., from 125 mL/d to 500 mL/d of wine for men, and from 62.5 mL/d up to 250 mL/d for women [35]; and e) heavy drinkers, i.e., those exceeding the limits set by the MDS, i.e., >250 mL/d and >500 mL/d of wine for women and men, respectively.

Adherence to a traditional MD was defined through the Mediterranean Diet Score (MDS) developed by Trichopoulou et al. [35], which was obtained by assigning one point to healthy foods (i.e., fruits and nuts, vegetables, legumes, fish, cereals, and monounsaturated to-saturated fat ratio) whose consumption was above the sex-specific medians of intake of the Moli-sani Study cohort; foods presumed to be detrimental (i.e., meat and dairy products) were scored positively if their consumption was below the population median. All other intakes received 0 points.

The ethanol component, defined as 1 point for consumption of 10–50 g/day for men and 5–25 g/day for women (all other intakes receiving 0 points), was excluded from the score to prevent collinearity with the exposure. The resulting modified MDS ranged from 0 to 8, with higher values indicating greater adherence.

### Ascertainment of Covariates

Information on sociodemographic factors, lifestyles and clinical variables were obtained by interviewer-administered questionnaires at study entry. Personal history of CVD and cancer was self-reported and confirmed by medical records and therapy. Participants were considered to have hypertension, hyperlipidemia or diabetes at baseline if they reported having been treated with disease-specific drugs. Leisure-time physical activity was expressed as daily energy expenditure in metabolic equivalent task-hours (MET-h/d) for sport, walking and gardening [36]. Height and weight were measured, and body mass index (BMI) was calculated as kg/m^2^ and grouped as normal weight (BMI ≤25), overweight (25>BMI <30), and obese (BMI ≥30). Participants were classified as never, current or former smokers (reported not having smoked at all over the previous 12 months or more). Education was based on the highest qualification attained and was categorized as up to lower secondary (≤8 years of study), upper secondary school (8–13 years of study) and postsecondary education (>13 years of study). Housing tenure was classified as rented, 1 dwelling ownership and >1 dwelling ownership. Additional covariates were considered for females only, and included the menopausal status, and use of hormone replacement therapy.

### Statistical Analysis

Characteristics of the study population were presented as absolute count and relevant percentages for categorical variables, or mean values and standard deviation (±SD) for continuous variables. Differences in the distribution of baseline covariates across categories of wine consumption were calculated using generalized linear models adjusted for age, sex and energy intake (GENMOD procedure for categorical variables, and GLM procedure for continuous variables in SAS software).

Multivariable linear regression analyses (PROC REG in SAS) were used to estimate the relation between total ethanol and wine intake (independent variables; categorical) with biological aging (dependent variable; continuous) and results were expressed as regression coefficients (β) with 95% confidence intervals (95%CI).

Potential confounders were selected based on previous literature and biological plausibility, rather than deferring to statistical criteria [37].

The multivariable models were adjusted for age, sex, energy intake, educational level, housing tenure, place of residence, leisure-time physical activity, smoking status, body mass index (categorical), history of CVD, cancer, diabetes, hypertension, and hyperlipidemia, as well as menopausal status, hormone replacement therapy, and oral contraceptive use (in females only), the MDS (excluding its alcohol component), and consumption of beer and spirits (for wine analyses only).

The association between wine (mL/d) or ethanol (g/d) consumption and biological aging was also investigated using restricted cubic spline functions, in the sample where former drinkers were removed (n = 21,748) [38]. Knots were placed at 50, 125 and 250 mL/d of wine or 15, 30 and 60 g/d of ethanol to allow comparability between the spline analyses and those presenting estimates across categories of wine or ethanol consumption.

Subgroup analyses were conducted to test the robustness of the findings and explore potential effect modification by sex, age groups (35–54; 55–64; and 65–99 years), adherence to a traditional MD, (categorized as low: MDS excluding ethanol < 6, or high: MDS excluding ethanol ≥6), and health status by excluding participants with a history of CVD, cancer, diabetes, hypertension, or hyperlipidemia (n = 13,998). To evaluate whether the effects of wine or ethanol consumption patterns differed across subgroups, appropriate multiplicative interaction terms were included in the multivariable models.

Missing values for covariates, i.e., history of CVD (n = 362), cancer (n = 80), diabetes (n = 277), hyperlipidemia (n = 207), hypertension (n = 155), menopausal status (n = 12), education (n = 16), housing (n = 40), smoking habits (n = 16), hormone replacement therapy (n = 1), leisure-time physical activity (n = 192) and BMI (n = 13), were handled using a multiple imputation technique (SAS PROC MI, followed by PROC MIANALYZE) to maximize data availability for all variables, avoid bias introduced by missing not-at-random data patterns and achieve robust results over different simulations (n = 10 imputed datasets). Data analysis was generated using SAS/STAT software, version 9.4 (SAS Institute Inc., Cary, NC, USA).

## Results

The sample under study consisted of 22,495 participants (47.9% males) with a mean CA of 55.6 years (SD ± 11.7 years), while BA was 54.9 years (SD ± 9.1 years), and Δage −0.7 years (SD ± 7.7 years). The proportions of abstainers, former drinkers, moderate drinkers, Mediterranean moderate drinkers, and heavy drinkers were 30.4%, 3.3%, 44.7%, 15.7%, and 5.9, respectively (Table 1).

**TABLE 1:** Characteristics of the study population across patterns of wine consumption (n = 22,495). (Italy, 2005–2010).

Variables	Whole sample	Patterns of wine consumption					p-value
		Abstainers	Former drinkers	Moderate drinkers	Mediterranean moderate drinkers	Heavy drinkers	
N of participants (%)	22,495	6,833 (30.4%)	747 (3.3%)	10,071 (44.7%)	3,526 (15.7%)	1,318 (5.8%)	​
Chronological age; CA (y; mean, SD)	55.6 (11.7)	54.2 (11.6)	57.7 (12.1)	55.5 (12.0)	57.4 (11.3)	57.6 (10.5)	<0.0001
Biological age; BA (y; mean, SD)	54.9 (9.1)	55.1 (9.1)	55.0 (9.9)	54.8 (9.2)	54.6 (8.8)	55.1 (8.5)	0.002
Biological aging (Δage y; mean, SD)	−0.7 (7.7)	−0.5 (7.6)	−0.6 (8.1)	−0.7 (7.7)	−1.0 (7.7)	−0.5 (7.3)	0.002
MDS (mean, SD)	4.0 (1.6)	3.9 (1.5)	4.3 (1.5)	4.0 (1.6)	4.1 (1.5)	4.0 (1.5)	<0.0001
Energy intake (mean, SD)	2080 (575)	1949 (535)	1974 (542)	2050 (546)	2,275 (551)	2,529 (578)	<0.0001
Males (%)	47.9	77.5	68.0	44.3	31.0	27.9	<0.0001
Education (%)	​	​	​	​	​	​	<0.0001
Up to lower school	52.4	54.2	55.6	47.2	57.9	66.3	​
Upper secondary	34.7	34.4	34.5	37.1	31.9	26.9	​
Postsecondary education	12.8	11.3	11.0	15.7	10.2	6.7	​
Missing data	0.1	0.1	0.0	0.1	0.1	0.1	​
Housing tenure (%)	​	​	​	​	​	​	<0.0001
Rent	8.7	9.4	10.4	8.5	7.7	7.8	​
One dwelling ownership	82.2	83.7	75.4	81.3	82.4	85.3	​
>1 dwelling ownership	8.9	6.7	14.2	10.0	9.7	6.8	​
Missing data	0.2	0.2	0.0	0.2	0.2	0.1	​
Place of residence (%)	​	​	​	​	​	​	<0.0001
Urban	67.5	64.2	83.4	70.4	65.3	58.8	​
Rural	32.5	35.8	16.6	29.6	34.7	41.2	​
Smoking status (%)	​	​	​	​	​	​	<0.0001
Non-smoker	49.6	60.7	55.6	48.0	38.3	31.6	​
Smokers	22.8	20.4	21.3	22.7	25.0	31.0	​
Former	27.5	18.8	23.2	29.2	36.7	37.3	​
Missing data	0.1	0.1	0.0	0.1	0.1	0.1	​
Body mass index (kg/m^2^)	​	​	​	​	​	​	<0.0001
BMI <25	27.4	29.5	27.3	27.5	24.8	22.3	​
25≤BMI >30	42.8	37.4	38.1	45.1	47.2	44.2	​
BMI ≥30	29.7	33.0	34.4	27.3	27.9	33.5	​
Missing data	0.1	0.1	0.1	0.1	0.0	0.0	​
Leisure-time PA (MET h-d; mean, SD)^a^	3.5 (4.0)	3.5 (3.5)	3.0 (3.2)	3.4 (4.0)	3.9 (4.5)	4.3 (5.2)	<0.0001
Menopausal status (%)	​	​	​	​	​	​	0.76
Yes	58.3	54.8	62.9	59.5	65.7	64.9	​
Missing data	0.1	0.1	0.0	0.0	0.0	0.1	​
Hormone replacement therapy (%)	​	​	​	​	​	​	<0.0001
Yes	5.8	4.5	7.3	7.0	5.7	8.1	​
Missing data	0.01	0.0	0.1	0.0	0.0	0.1	​
Oral contraception use (%)	​	​	​	​	​	​	​
Yes	27.7	28.4	25.2	28.0	24.8	24.7	0.48
Missing data	0.02	0.1	0.0	0.0	0.1	0.1	​
Cardiovascular disease (%)	​	​	​	​	​	​	0.02
Yes	5.2	4.6	6.7	5.5	5.6	4.2	​
Missing data	1.6	1.7	1.1	1.5	1.8	2.0	​
Cancer (%)	​	​	​	​	​	​	0.02
Yes	3.5	3.5	5.2	3.5	3.9	2.0	​
Missing data	0.4	0.3	0.5	0.4	0.3	0.4	​
Diabetes (%)	​	​	​	​	​	​	0.006
Yes	4.9	4.9	7.6	4.9	4.6	4.0	​
Missing data	1.2	1.3	2.1	1.1	1.2	1.4	​
Hypertension (%)	​	​	​	​	​	​	0.005
Yes	28.6	28.3	33.3	28.4	28.4	29.1	​
Missing data	0.7	0.6	0.4	0.6	0.9	1.4	​
Hyperlipidaemia (%)	​	​	​	​	​	​	<0.0001
Yes	7.8	7.0	8.8	8.1	8.7	6.4	​
Missing data	0.9	0.7	2.3	1.0	0.9	1.2	​

MDS, mediterranean diet score not including ethanol component; MET-h, metabolic equivalent of task hour; PA, physical activity.

P-values were obtained was adjusted for age and sex.

^a^ Available for 22,303 participants.

CA differed between groups, with Mediterranean moderate and heavy drinkers being older than abstainers and moderate drinkers. BA showed smaller differences, with Mediterranean moderate drinkers having a slightly lower BA. Biological aging was lowest among Mediterranean moderate drinkers (Table 1).

Abstainers and former drinkers included a higher proportion of males (77.5% and 68.0%, respectively) compared to Mediterranean moderate (31.0%) and heavy drinkers (27.9%). Education levels and housing tenure also varied among groups, with heavy drinkers having a higher prevalence of lower education attainment. Multiple property ownership was lower among both heavy drinkers and abstainers compared with the other drinking categories. Mediterranean moderate and heavy drinkers reported higher energy intake, leisure-time physical activity, a greater percentage of former smokers, and lower BMI. Chronic conditions such as diabetes, hypertension, hyperlipidemia, CVD, and cancer showed variation across groups (Table 1).

In multivariable-adjusted regression analyses controlling for known risk factors and using abstainers as the reference category, Mediterranean moderate drinkers showed a small difference toward a slower increase in Δage (β = −0.22 years; 95% CI: −0.47 to 0.03) Heavy drinking showed a similarly small difference in the opposite direction (β = 0.20 years; 95% CI: −0.16–0.56), with wide confidence intervals for both estimates, spanning from a modest change to little or no difference.

Men classified as Mediterranean moderate wine drinkers had lower Δage compared to abstainers (β = −0.39; 95%CI: −0.78 to −0.01), whereas no differences in Δage were observed across wine consumption patterns in women. However, the formal test for interaction did not support sex-specific differences (p for interaction = 0.21); as an omnibus test across categories, it may have limited sensitivity to detect differences confined to a single category (Table 2).

**TABLE 2:** Association between patterns of wine consumption and biological aging in the Moli-sani Study cohort and in men and women separately. (Italy, 2005–2010).

*Whole sample (n = 22,495)*	*Biological aging (Δage)*						
Pattern of consumption	N of participants (%)	Mean (SD)^a^	p-value	β (95%CI)^b^	p-value^b^	β (95%CI)^c^	p-value^c^
Abstainers	6,833 (30.4)	−0.5 (7.6)	0.004	-Ref-	‒	-Ref-	‒
Former drinkers	747 (3.3)	−0.5 (8.1)		−0.08 (−0.53 to 0.37)	0.73	−0.12 (−0.55 to 0.32)	0.60
Moderate drinkers	10,071 (44.8)	−0.7 (7.7)		−0.23 (−0.42 to −0.04)	0.02	−0.08 (−0.27 to 0.10)	0.37
Mediterranean moderate drinkers	3,526 (15.7)	−0.9 (7.7)		−0.47 (−0.73 to −0.22)	0.0003	−0.22 (−0.47 to 0.03)	0.086
Heavy drinkers	1,318 (5.8)	−0.4 (7.3)		0.05 (−0.31–0.41)	0.77	0.20 (−0.16–0.56)	0.29

*Men (n = 10,769)*	*Biological aging (Δage)*						
Pattern of consumption	N of participants (%)	Mean (SD)^a^	p-value	β (95%CI)^b^	p-value^b^	β (95%CI)^c^	p-value^c^
Abstainers	1,534 (14.3)	−0.4 (8.0)	<0.0001	-Ref-	‒	-Ref-	‒
Former drinkers	239 (2.2)	−0.1 (9.0)		0.26 (−0.56–1.08)	0.53	0.17 (−0.63–0.97)	0.67
Moderate drinkers	5,614 (52.1)	−0.7 (7.8)		−0.33 (−0.67 to 0.01)	0.06	−0.27 (0.61–0.06)	0.10
Mediterranean moderate drinkers	2,432 (22.6)	−0.8 (7.7)		−0.53 (−0.92 to −0.14)	0.007	−0.39 (−0.78 to −0.01)	0.048
Heavy drinkers	950 (8.8)	−0.4 (7.4)		−0.09 (−0.58 to 0.39)	0.72	0.03 (−0.47–0.53)	0.91

*Women (n = 11,726)*	*Biological aging (Δage)*						
Pattern of consumption	N of participants (%)	Mean (SD)^a^	p-value	β (95%CI)^b^	p-value^b^	β (95%CI)^c^	p-value^c^
Abstainers	5,299 (45.2)	−0.6 (7.5)	<0.0001	-Ref-	‒	-Ref-	‒
Former drinkers	508 (4.3)	−0.9 (7.7)		−0.26 (−0.80 to 0.28)	0.34	−0.28 (−0.80 to 0.24)	0.29
Moderate drinkers	4,457 (38.0)	−0.8 (7.6)		−0.19 (−0.42 to 0.05)	0.12	0.07 (−0.16–0.31)	0.54
Mediterranean moderate drinkers	1,094 (9.3)	−1.0 (7.6)		−0.46 (−0.85 to −0.08)	0.02	−0.11 (−0.49 to 0.26)	0.55
Heavy drinkers	368 (3.2)	−0.2 (7.0)		0.31 (−0.31–0.94)	0.32	0.46 (−0.14–1.07)	0.13

^a^ Means and p-values were obtained from generalized linear models adjusted for age, sex (not for analyses by sex), energy intake, and consumption of beer and spirits.

^b^ Regression coefficients β (95%CI), expressed in years, were obtained from a multivariable model controlled for age, sex (not for analyses by sex), energy intake, and consumption of beer and spirits.

^c^ Regression coefficients β (95%CI) were obtained from a multivariable model controlled for age, sex (not for analyses by sex), energy intake, educational level, housing, place of residence, leisure-time physical activity, smoking habit, body mass index, history of cardiovascular disease, cancer, diabetes, hypertension, hyperlipidaemia, the Mediterranean Diet Score deprived of its ethanol component, beer and spirit consumption, menopausal status, hormone replacement therapy, and oral contraception use (for women only).

**FIGURE 1:**
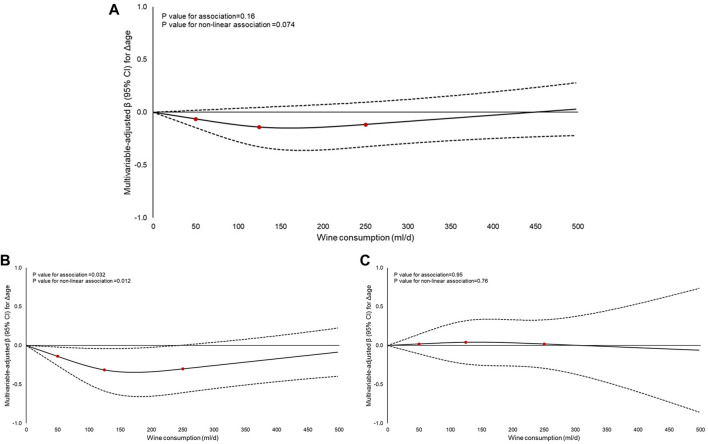
Dose-response analysis of the association of wine consumption (mL/d) with biological aging in: **(A)** the overall Moli-sani Study cohort (n = 21,748); **(B)** men (n = 10,530); and **(C)** women (n = 11,218). (Italy, 2005–2010). Regression coefficients β (95%CI) were obtained from a multivariable-adjusted model controlled for age, sex (not in analyses by sex), energy intake, educational level, housing, place of residence, leisure-time physical activity, smoking habit, body mass index, history of cardiovascular disease, cancer, diabetes, hypertension, hyperlipidemia, the Mediterranean Diet Score deprived of its ethanol component, beer and spirit consumption, menopausal status, hormone replacement therapy, and oral contraception use (for women only). Wine consumption was modelled as a continuous exposure. Zero value was used as the reference. Former drinkers were excluded from this analysis. Three knots were placed at the at 50, 125 and 250 mL/d of wine consumption distribution. The dashed lines indicate 95% confidence bands.

These associations were also explored in dose-response analyses, which suggested a non-linear, J-shaped relationship between wine consumption (mL/d) and Δage in the overall sample (Figure 1A), with a weak overall association, the lowest estimates at moderate intakes, and wide uncertainty bands, consistent with the category-based results.

In sex-stratified analyses, a J-shaped association was observed in men (Figure 1B), whereas the relationship was virtually null in women (Figure 1C). In men, the nadir was identified at 172 mL/d (difference = −0.34; 95% CI: −0.66 to – 0.03), with protective effects extending up to 240 mL/day (difference = −0.31; 95% CI: −0.62 to −0.00046).

The association between Mediterranean moderate wine consumption and Δage did not differ across levels of adherence to the MD (β in the low MD adherence group: = −0.25; 95% CI: −0.53 to 0.02 vs. abstainers; and β in the high MD adherence group: = −0.08; 95% CI: −0.69 to 0.54 vs. abstainers), and this was confirmed also by a formal test of interaction (p-value for interaction = 0.82) (Table 3).

**TABLE 3:** . Association between patterns of wine consumption and biological aging in the Moli-sani Study cohort by level of adherence to a traditional Mediterranean Diet (MD). (Italy, 2005–2010).

*Low MD adherence (n = 18,481)*	*Biological aging (Δage)*						
Pattern of consumption	N of participants (%)	Mean (SD)^a^	p-value	β (95%CI)^b^	p-value^b^	β (95%CI)^c^	p-value^c^
Abstainers	5,722 (31.0)	−0.6 (7.7)	0.22	-Ref-	‒	-Ref-	‒
Former drinkers	573 (3.1)	−0.7 (8.2)		−0.17 (−0.69 to 0.33)	0.51	−0.21 (−0.71 to 0.30)	0.40
Moderate drinkers	8,221 (44.5)	−0.6 (7.7)		−0.23 (−0.44 to −0.02)	0.031	−0.08 (−0.29 to 0.13)	0.45
Mediterranean moderate drinkers	2,876 (15.6)	−0.8 (7.7)		−0.47 (−0.76 to −0.19)	0.0009	−0.25 (−0.53 to 0.02)	0.074
Heavy drinkers	1,089 (5.9)	−0.3 (7.3)		0.01 (−0.38–0.41)	0.95	0.14 (−0.26–0.54)	0.49

*High MD adherence (n = 4,014)*	*Biological aging (Δage)*						
Pattern of consumption	N of participants (%)	Mean (SD)^a^	p-value	β (95%CI)^b^	p-value^b^	β (95%CI)^c^	p-value^c^
Abstainers	1,111 (27.7)	−0.9 (7.2)	0.56	-Ref-	‒	-Ref-	‒
Former drinkers	174 (4.3)	−0.7 (7.9)		0.23 (−0.72–1.18)	0.63	0.17 (−0.76–1.09)	0.72
Moderate drinkers	1,850 (46.1)	−1.0 (7.6)		−0.22 (−0.68 to 0.24)	0.35	−0.11 (−0.57 to 0.34)	0.63
Mediterranean moderate drinkers	650 (16.2)	−1.2 (7.6)		−0.46 (−1.07 to 0.15)	0.14	−0.08 (−0.69 to 0.54)	0.80
Heavy drinkers	229 (5.7)	−0.5 (7.5)		0.21 (−0.65–1.08)	0.63	0.46 (−0.42–1.33)	0.31

Low and high adherence to the Mediterranean Diet Score (excluding its ethanol component) were defined as values below 6 or equal to or greater than 6, respectively.

^a^ Means and p-values were obtained from generalized linear models adjusted for age, sex (not for analyses by sex), energy intake, and consumption of beer and spirits.

^b^ Regression coefficients β (95%CI) were obtained from a multivariable model controlled for age, sex (not for analyses by sex), energy intake, and consumption of beer and spirits.

^c^ Regression coefficients β (95%CI) were obtained from a multivariable model controlled for age, sex, energy intake, educational level, housing, place of residence, leisure-time physical activity, smoking habit, body mass index, history of cardiovascular disease, cancer, diabetes, hypertension, hyperlipidaemia, beer and spirit consumption, menopausal status, hormone replacement therapy, and oral contraception use (for women only).

Age-stratified analyses showed no difference in the association between wine consumption and Δage among participants aged 35–54 years (Supplementary Figure S2a) and in elderly participants (Supplementary Figure S2c). In middle-aged individuals (Supplementary Figure S2b), a modest inverse association was observed at moderate intake (∼100–150 mL/day), though confidence intervals widened at higher consumption and non-linear trends were borderline, indicating a weak and uncertain effect.

Results from analyses conducted in a healthy sample free from major health conditions were consistent with those observed in the whole study sample (Supplementary Figure S3a).

A J-shaped association was observed among healthy men (Supplementary Figure S3b), similar to that seen in the general male population, whereas no association was found in healthy women (Supplementary Figure S3c).

**FIGURE 2:**
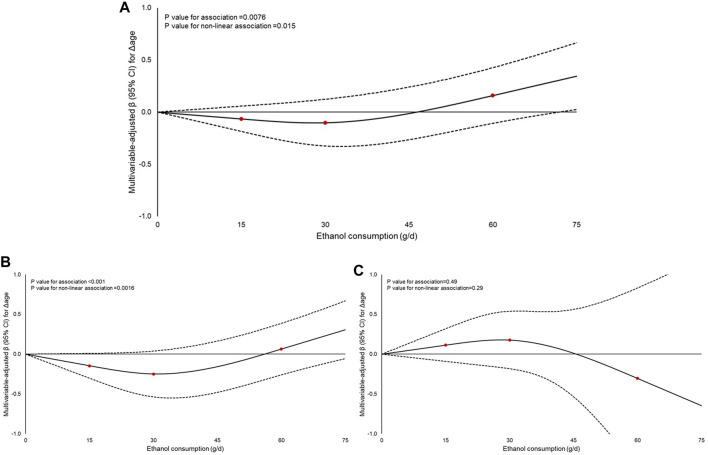
Dose-response analysis of the association of ethanol consumption (g/d) with biological aging in: **(A)** the overall Moli-sani Study cohort (n = 21,748); **(B)** men (n = 10,530); and **(C)** women (n = 11,218). (Italy, 2005–2010). Regression coefficients β (95%CI) were obtained from a multivariable-adjusted model controlled for age, sex (not in analyses by sex), energy intake, educational level, housing, place of residence, leisure-time physical activity, smoking habit, body mass index, history of cardiovascular disease, cancer, diabetes, hypertension, hyperlipidemia, the Mediterranean Diet Score deprived of its ethanol component, menopausal status, hormone replacement therapy, and oral contraception use (for women only). Ethanol consumption was modelled as a continuous exposure. Zero value was used as the reference. Former drinkers were excluded from this analysis. Three knots were placed at the at 15, 30 and 60 g/d of ethanol consumption distribution. The dashed lines indicate 95% confidence bands.

Regarding ethanol consumption, none of the drinking patterns were associated with Δage overall or by sex, although an upward trend was observed in men classified as heavy drinkers compared to abstainers (β = 0.46 years; 95% CI: −0.06–0.98) (Supplementary Table S1). Dose-response analyses indicated that low to moderate ethanol intakes (0–30 g/day) were linked to either slight decreases or negligible changes in Δage, suggesting little effect on biological aging. In contrast, higher intakes (>30 g/day) showed a progressively positive association, with β estimates pointing to accelerated biological aging at levels above 60 g/day (Figure 2A). Similar patterns were observed in men (Figure 2B) but not in women (Figure 2C).

Analyses using MDS and its individual components showed that the MDS, consumption of vegetables, the monounsaturated-to-saturated fat ratio, and meat consumption were inversely associated with Δage in a dose-response manner, with evidence of a non-linear relationship (p-values for non-linearity <0.050) (Supplementary Figure S4, 5B, 6B, 6C). In contrast, consumption of fruits, cereals, legumes, fish, and dairy were not associated with Δage (Supplementary Figures S5, S6).

## Discussion

Our findings from a large population-based cohort in Southern Italy indicate that moderate wine consumption, as defined by a traditional MD, is associated with slower biological aging in men. A dose-response relationship was also observed, suggesting that moderate intake, consistent with a traditional MD, may positively influence aging trajectories. On average, men consuming about 200 mL of wine per day (roughly 1.5–2 glasses) were approximately 0.4 years biologically younger than non-drinkers.

The J-shaped relationship observed in men is consistent with prior work reporting a similar pattern between cardiovascular mortality and alcohol intake [39], suggesting that even low doses of alcohol may have protective effects that do not diminish at higher intakes.

Sex related differences in alcohol’s health effects may explained by i.e., variations in metabolism and hormones [40]. Women generally have lower alcohol dehydrogenase levels, resulting in greater alcohol absorption and stronger effects at lower doses. Hormonal factors may also contribute, though evidence from human studies on estrogen-related influences is inconsistent [41].

In our study, the inverse association between wine intake and biological aging appeared stronger in middle-aged participants. This may reflect age-related declines in liver function and alcohol-metabolizing enzymes, which slow alcohol processing and can increase related health risks [42].

Our analysis on the MDS and its individual components revealed that the J-shaped association was not limited to wine consumption, but also extended to other MD components, such as vegetables and the ratio of monounsaturated to saturated fats. These findings support our main analysis on wine, suggesting that wine is not the only dietary component showing this type of J-shaped association with biological aging. The counterintuitive results for meat consumption could reflect a protective role of animal proteins, particularly in preserving muscle mass and function during aging [43], as well as providing essential micronutrients such as vitamin B12, iron, and zinc, which are critical for healthy aging [44].

To date, previous studies have primarily focused on total ethanol intake, often in the context of heavy drinking or alcohol use disorders, and apparently no research has specifically examined Mediterranean populations [24–26].

Large scale studies using UK Biobank and Generation Scotland data found no evidence of an effect of alcohol consumption on accelerated biological aging, as assessed by measures of brain and epigenetic aging [45]. A Mendelian randomization study in the UK Biobank suggested that alcohol intake may shorten telomeres, but observational analyses showed this only for high consumption (≥17–29 units/week, about two and a half to four glasses per day), not for moderate drinking [24]. Also, in these analyses, overall diet quality was not included as an adjustment factor, thereby overlooking drinking habits in the context of the whole diet.

The Framingham Heart Study found that alcohol consumption, especially at higher levels and from liquor, was linked to faster epigenetic aging in mid-to-late adulthood. Alcohol was treated as a continuous exposure, with hazard ratios reported per unit increase in consumption, and drinking patterns considered only at-risk or heavy drinkers, while moderate consumption categories were not included [25].

In the Coronary Artery Risk Development in Young Adults Study, cumulative liquor and total alcohol consumption, as well as recent binge drinking, were associated with increased GrimAge acceleration, a second-generation marker of epigenetic age. Interestingly, beer showed only marginal associations, whereas wine showed no association with accelerated aging [26].

It is worth noting that all these studies analyzed alcohol consumption without distinguishing moderate intake thresholds-levels typically linked to cardiovascular benefits, and only one considered wine specifically as the exposure [25]. This distinction may be critical, as patterns of alcohol consumption, including beverage type, frequency and consumption with food, may have different associations with human health [46].

There are several potential mechanisms that can explain our findings. Wine is a major source of polyphenols which have been implicated in reducing inflammation, oxidative stress, and improving metabolic function, all mechanisms involved in aging, and specifically reflected in our biological age measure [13].

It is worth noting that, in our analyses, ethanol intake was not associated with slower biological aging, which points to non-ethanol components (e.g., polyphenols) as a more plausible explanation for the associations observed with wine, in line with previous evidence observed in the Moli-sani cohort [10]. Additionally, previous studies have shown that the protective associations of alcohol with certain health outcomes are more pronounced among wine drinkers, suggesting that these benefits may stem from components other than ethanol [16].

Finally, it is worth mentioning that previous cohort studies examining the association between alcohol consumption and various markers of biological aging have been conducted in non-Mediterranean populations. Importantly, alcohol metabolism is known to vary considerably across ethnic groups due to genetic differences in enzymes such as alcohol dehydrogenase and aldehyde dehydrogenase [47], which may influence drinking habits [48], as well as the physiological effects of alcohol and its relationship with aging-related outcomes [49].

Notably, in our study, the association between wine consumption and biological aging was similar across levels of MD adherence, potentially reflecting wine-specific components or consumption patterns rather than a broader dietary effect.

### Strengths and Limitations

To our knowledge, this is the first study to examine the association between patterns of wine consumption and a blood-based measure of biological aging. We employed an innovative, deep learning–based measure of biological aging that integrates a large number of circulating biomarkers, tagging different domains. Additional strengths of our study include its large sample size and comprehensive assessment of diet and other risk factors, which helps to partially reduce potential confounding.

Our study, however, has several limitations. First, as an observational study, it cannot establish causality and cannot fully rule out residual or unmeasured confounding. Its cross-sectional design also limits the ability to determine the directionality of the observed associations. However, reverse causality is implausible in this context, as it is highly unlikely that a BA profile would determine wine consumption patterns. Additionally, dietary data, including alcohol and wine consumption, were based on self-reported information collected through an FFQ. This information is prone to recall bias, social desirability bias, and under- or over-reporting, particularly for binge or irregular drinking patterns [50]. In addition, FFQs may introduce measurement error due to difficulties in estimating portion sizes, variability in alcohol content of beverages, and potential inadequacies in food composition databases. However, these issues were partially mitigated by excluding participants with implausible energy intakes and by adjusting for total energy intake [51]. Furthermore, the FFQ used in this study has been widely employed in previous research and has demonstrated predictive validity for several health outcomes [52].

Finally, our data were gathered from an adult cohort in a Southern Italian region, which may limit the generalizability of our findings to other populations.

### Conclusions

We found that moderate wine consumption, as defined by a traditional MD, is associated with slower blood-based biological aging in men of a well-characterized Southern Italian cohort. The observed dose-response relationship indicates potential benefits for both men and women. In contrast, ethanol intake did not show comparable associations with biological aging, suggesting that the observed effects are more likely attributable to wine-specific components such as polyphenols.

Overall, our study provides novel evidence that moderate wine consumption following the traditional MD definition may slow biological aging in men, emphasizing the relevance of both beverage type and dietary pattern. Further longitudinal and mechanistic studies are needed to confirm these associations and clarify underlying pathways.
