# Correction: Moderate Wine Consumption, Defined by the Mediterranean Diet, Is Associated With Delayed Biological Aging in Men From the Moli-Sani Study

**DOI:** 10.3389/ijph.2026.1609829

**Published:** 2026-04-27

**Authors:** Simona Esposito, Augusto Di Castelnuovo, Simona Costanzo, Alessandro Gialluisi, Antonietta Pepe, Emilia Ruggiero, Amalia De Curtis, Sara Magnacca, Mariarosaria Persichillo, Francesc Casanovas-Garriga, Chiara Cerletti, Maria Benedetta Donati, Giovanni de Gaetano, Licia Iacoviello, Marialaura Bonaccio

**Affiliations:** 1 Research Unit of Epidemiology and Prevention, IRCCS Neuromed, Pozzilli, Isernia, Italy; 2 Department of Medicine and Surgery, LUM University, Bari, Italy; 3 Department of Medicine and Surgery, Research Center in Epidemiology and Preventive Medicine (EPIMED), University of Insubria, Varese, Italy; 4 Department of Internal Medicine, Hospital Clínic de Barcelona, University of Barcelona (UB), Barcelona, Spain; 5 Institut de Recerca en Nutrició i Seguretat Alimentaria (INSA-UB), University of Barcelona (UB), Barcelona, Spain; 6 Institut d’Investigacions Biomèdiques August Pi i Sunyer (IDIBAPS), Barcelona, Spain

**Keywords:** biological aging, mediterranean diet, neurological decline, prevention, wine consumption

The funder the Italian Ministry of Health project GR-2021-12375341 to AG, was erroneously omitted.

The original article has been updated.

