# Supplementary material for: Moderate Wine Consumption, Defined by the Mediterranean Diet, Is Associated With Delayed Biological Aging in Men From the Moli-sani Study
Source: Int J Public Health. 2026 Mar 16;71:1609410. doi: 10.3389/ijph.2026.1609410 (PMC13033601; doi:10.3389/ijph.2026.1609410)
Supplement: Supplementary file 1 [file Supplementaryfile1.docx]

**Supplementary Figure 1.** Flowchart for selection of study participants from the Moli-sani Study, 2005-2010.

**
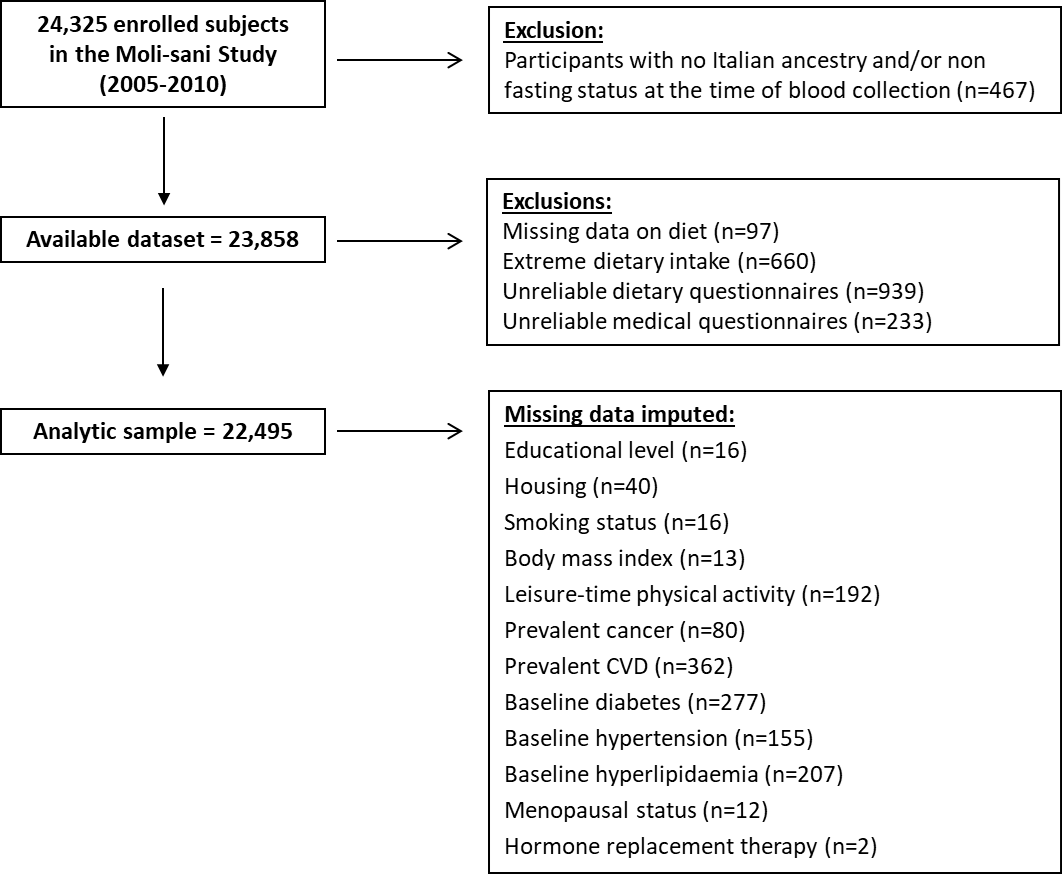
**

**Supplementary Table 1.** Association between patterns of ethanol consumption and biological aging in the Moli-sani Study cohort and in men and women separately.

| *Whole sample (n=22,495)* | *Biological aging (∆age)* | | | | | | |
| --- | --- | --- | --- | --- | --- | --- | --- |
| **Pattern of consumption** | N of  participants (%) | Mean (SD)* | p-value | β (95%CI)^1^ | p-value^1^ | β (95%CI)^2^ | p-value^2^ |
| Abstainers | 6,202 (27.6%) | -0.5 (7.6) | 0.002 | -Ref- | ‒ | -Ref- | ‒ |
| Former drinkers | 747 (3.3%) | - 0.6 (8.1) |  | -0.10 (-0.55 to 0.36) | 0.67 | -0.13 (-0.57 to 0.30) | 0.55 |
| Moderate drinkers | 10,626 (47.2%) | -0.8 (7.7) |  | -0.29 (-0.48 to -0.09) | 0.004 | -0.09 (-0.29 to 0.09) | 0.33 |
| Mediterranean  moderate drinkers | 3,684 (16.4%) | -0.8 (7.7) |  | -0.36 (-0.62 to -0.11) | 0.006 | -0.09 (-0.35 to 0.17) | 0.49 |
| Heavy drinkers | 1,236 (5.5%) | -0.2 (7.4) |  | 0.12 (-0.25 to 0.51) | 0.52 | 0.32 (-0.06 to 0.70) | 0.10 |
|  |  |  |  |  |  |  |  |
| *Men (n=10,769)* | *Biological aging (∆age)* | | | | | | |
| **Pattern of consumption** | N of  participants (%) | Mean (SD)* | p-value | β (95%CI)^1^ | p-value^1^ | β (95%CI)^2^ | p-value^2^ |
| Abstainers | 1,250 (11.6%) | -0.6 (8.0) | <0.0001 | -Ref- | ‒ | -Ref- |  |
| Former drinkers | 239 (2.2%) | - 0.2 (9.0) |  | 0.38 (-0.45 to 1.21) | 0.37 | 0.24 (-0.57 to 1.06) | 0.56 |
| Moderate drinkers | 5,727 (53.2%) | -0.7 (7.8) |  | -0.23 (-0.60 to 0.14) | 0.22 | -0.12 (-0.48 to 0.23) | 0.50 |
| Mediterranean  moderate drinkers | 2,586 (24.0%) | -0.8 (7.7) |  | -0.39 (-0.79 to 0.02) | 0.06 | -0.18 (-0.58 to 0.23) | 0.39 |
| Heavy drinkers | 967 (9.0%) | 0.03 (7.5) |  | 0.27 (-0.23 to 0.78) | 0.28 | 0.46 (-0.06 to 0.98) | 0.085 |
|  |  |  |  |  |  |  |  |
| *Women (n=11,726)* | *Biological aging (∆age)* | | | | | | |
| **Pattern of consumption** | N of  participants (%) | Mean (SD)* | p-value | β (95%CI)^1^ | p-value^1^ | β (95%CI)^2^ | p-value^2^ |
| Abstainers | 4,952 (42.2%) | -0.6 (7.5) | <0.0001 | -Ref- | ‒ | -Ref- | ‒ |
| Former drinkers | 508 (4.3%) | - 0.9 (7.7) |  | -0.31 (-0.85 to 0.22) | 0.25 | -0.32 (-0.84 to 0.20) | 0.23 |
| Moderate drinkers | 4,899 (41.8%) | -0.9 (7.6) |  | -0.32 (-0.55 to -0.09) | 0.007 | -0.01 (-0.23 to 0.22) | 0.95 |
| Mediterranean  moderate drinkers | 1,098 (9.4%) | -0.7 (7.7) |  | -0.20 (-0.59 to 0.18) | 0.30 | 0.17 (-0.20 to 0.55) | 0.36 |
| Heavy drinkers | 269 (2.3%) | -0.7 (6.9) |  | -0.20 (-0.92 to 0.52) | 0.59 | 0.09 (-0.61 to 0.79) | 0.80 |

*Means and p-values were obtained from generalized linear models adjusted for age, sex (not for analyses by sex), energy intake.

^1^ Regression coefficients β (95%CI) were obtained from a multivariable model controlled for age, sex (not for analyses by sex), and energy intake.

^2^ Regression coefficients β (95%CI) were obtained from a multivariable model controlled for age, sex (not for analyses by sex), energy intake, educational level, housing, place of residence, leisure-time physical activity, smoking habit, body mass index, history of cardiovascular disease, cancer, diabetes, hypertension, hyperlipidaemia, the Mediterranean Diet Score deprived of its ethanol component, menopausal status, hormone replacement therapy, and oral contraception use (for women only).

**Supplementary Figure 2.** Multivariable dose-response association between wine consumption (mL/d) with biological aging in: a) participants aged 35-54 years; b) aged 55-64 years; and c) aged 65-99 years in the Moli-sani Study cohort, estimated using restricted cubic spline Cox regression (3 knots at 50, 125 and 250 mL/d). The reference value for hazard ratios was wine intake = 0. Dashed lines indicate 95% confidence intervals. The model was adjusted for sex, age, energy intake, educational level, housing, place of residence, leisure-time physical activity, smoking habit, body mass index, history of cardiovascular disease, cancer, diabetes, hypertension, hyperlipidaemia, the Mediterranean Diet Score deprived of its ethanol component, beer and spirit consumption menopausal status, hormone replacement therapy, and oral contraception use (for women only and limited to the first two age groups).


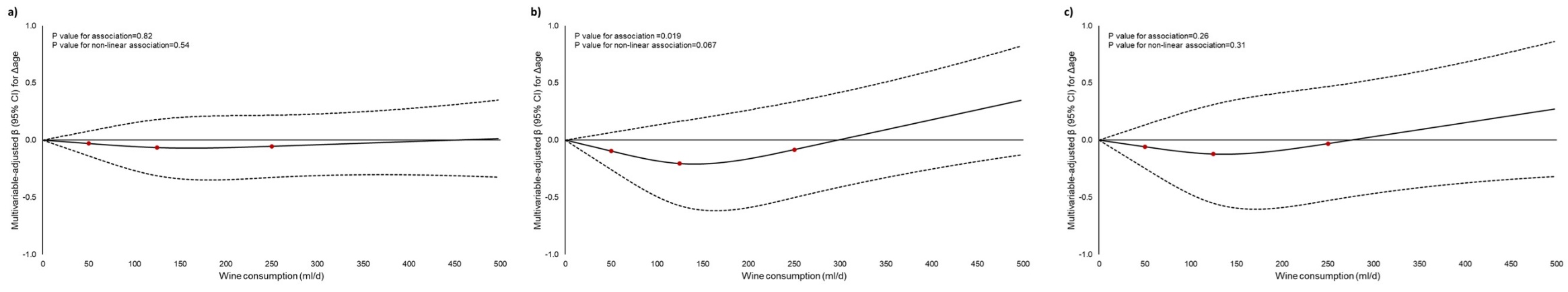


**Supplementary Figure 3.** Multivariable dose-response associations between wine consumption (mL/d) and biological aging were examined in: (a) a healthy sample free from a history of CVD, cancer, diabetes, hypertension, or hyperlipidemia (n=13,998) from the Moli-sani Study cohort; (b) healthy men (n=6671); and (c) healthy women (n=7327). Associations were estimated using restricted cubic spline Cox regression with 3 knots at 50, 125, and 250 mL/d. The reference value for hazard ratios was wine intake = 0. Dashed lines indicate 95% confidence intervals. Models were adjusted for sex, age, energy intake, educational level, housing, place of residence, leisure-time physical activity, smoking habit, body mass index, the Mediterranean Diet Score excluding its ethanol component, beer and spirit consumption, menopausal status, hormone replacement therapy, and oral contraception use (for women only).


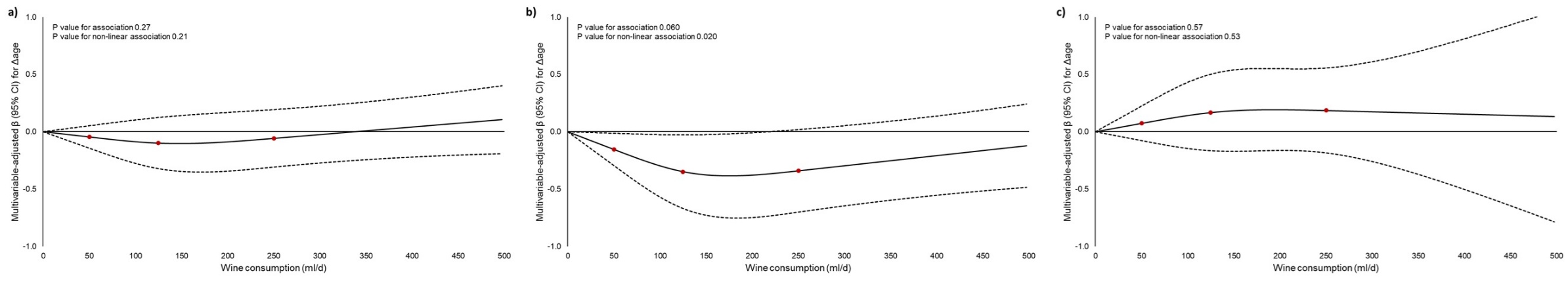


**Supplementary Figure 4.** Multivariable dose-response association between the Mediterranean Diet Score (MDS) with biological aging in the Moli-sani Study cohort (n=22,495), estimated using restricted cubic spline Cox regression (3 knots at the 5th, 50th, and 95th percentiles of each dietary exposure). Reference value for hazard ratios was set at MDS=0. Dashed lines indicate 95% confidence intervals. The model was adjusted for sex, age, energy intake, educational level, housing, place of residence, leisure-time physical activity, smoking habit, body mass index, history of cardiovascular disease, cancer, diabetes, hypertension, hyperlipidaemia, menopausal status, hormone replacement therapy, and oral contraception use (for women only).

**
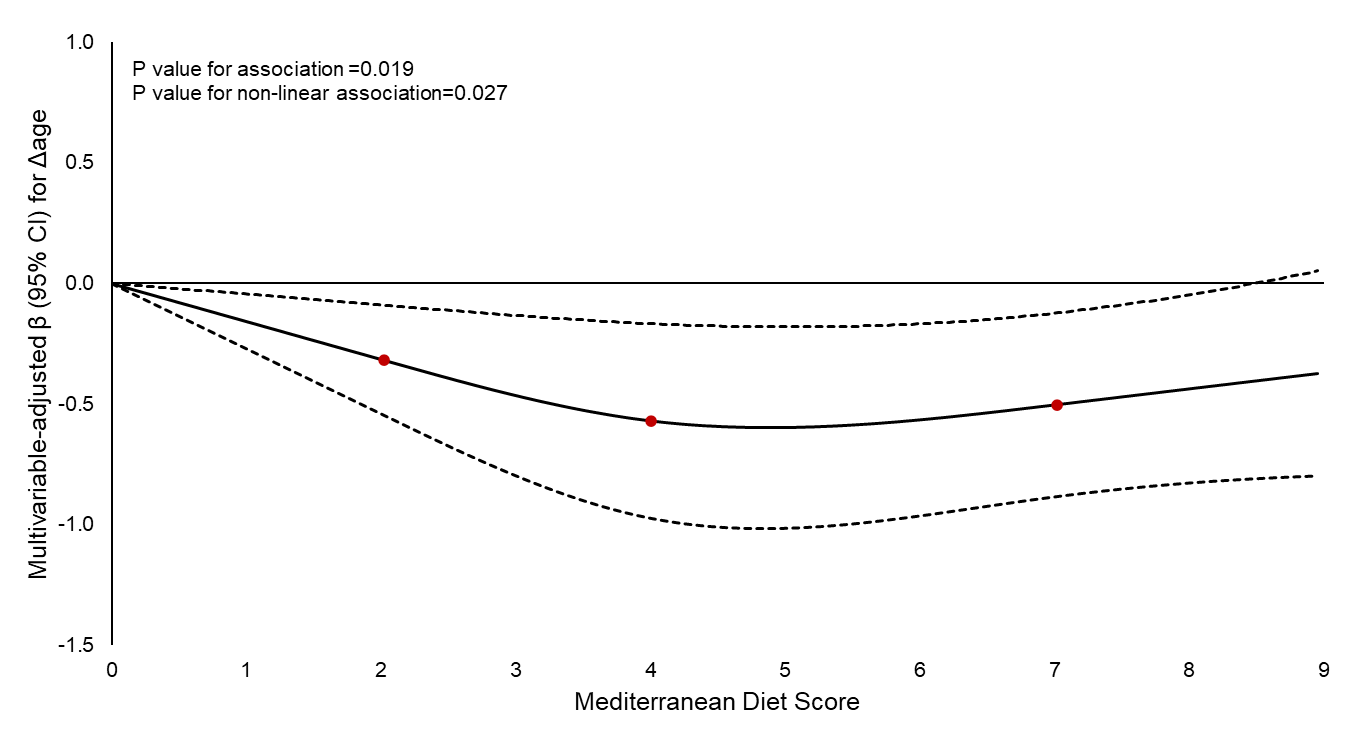
**

**Supplementary Figure 5.** Multivariable dose-response association between a) fruit consumption; b) vegetables consumption; c) cereals consumption; and d) fish consumption with biological aging in the Moli-sani Study cohort (n=22,495), estimated using restricted cubic spline Cox regression (3 knots at the 5th, 50th, and 95th percentiles of each dietary exposure). Reference value for hazard ratios was set at 0. Dashed lines indicate 95% confidence intervals. The model was adjusted for sex, age, energy intake, educational level, housing, place of residence, leisure-time physical activity, smoking habit, body mass index, history of cardiovascular disease, cancer, diabetes, hypertension, hyperlipidaemia, menopausal status, hormone replacement therapy, and oral contraception use (for women only). Each component of the MDS was analysed while controlling for the overall MDS score with that component removed.


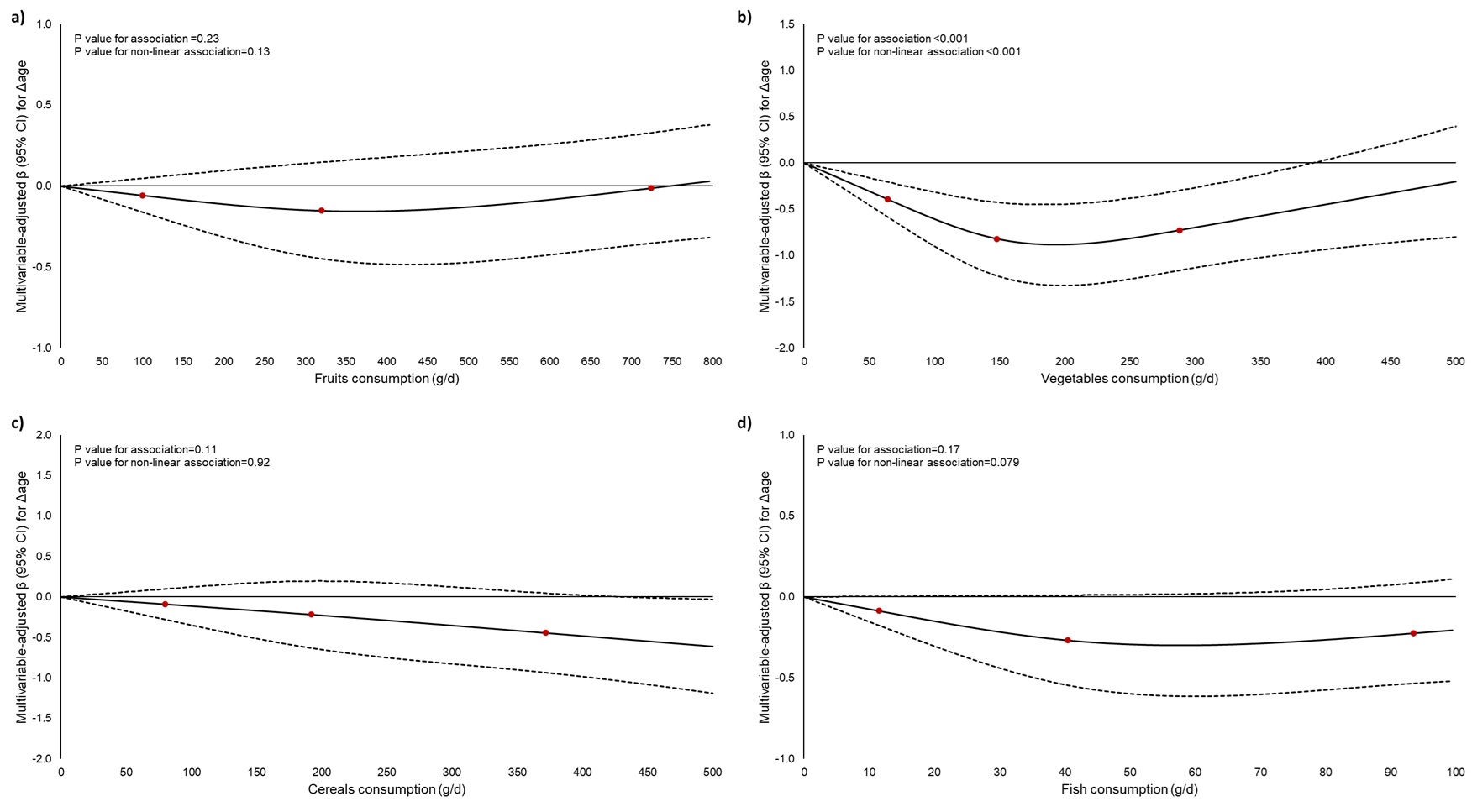


**Supplementary Figure 6.** Multivariable dose-response association between a) legumes consumption; b) monounsaturated to saturated fat ratio; c) milk and dairy products consumption; and d) meat and meat products consumption, with biological aging in the Moli-sani Study cohort (n=22,495), estimated using restricted cubic spline Cox regression (3 knots at the 5th, 50th, and 95th percentiles of each dietary exposure). Reference value for hazard ratios was set at 0. Dashed lines indicate 95% confidence intervals. The model was adjusted for sex, age, energy intake, educational level, housing, place of residence, leisure-time physical activity, smoking habit, body mass index, history of cardiovascular disease, cancer, diabetes, hypertension, hyperlipidaemia, menopausal status, hormone replacement therapy, and oral contraception use (for women only). Each component of the MDS was analysed while controlling for the overall MDS score with that component removed.

**
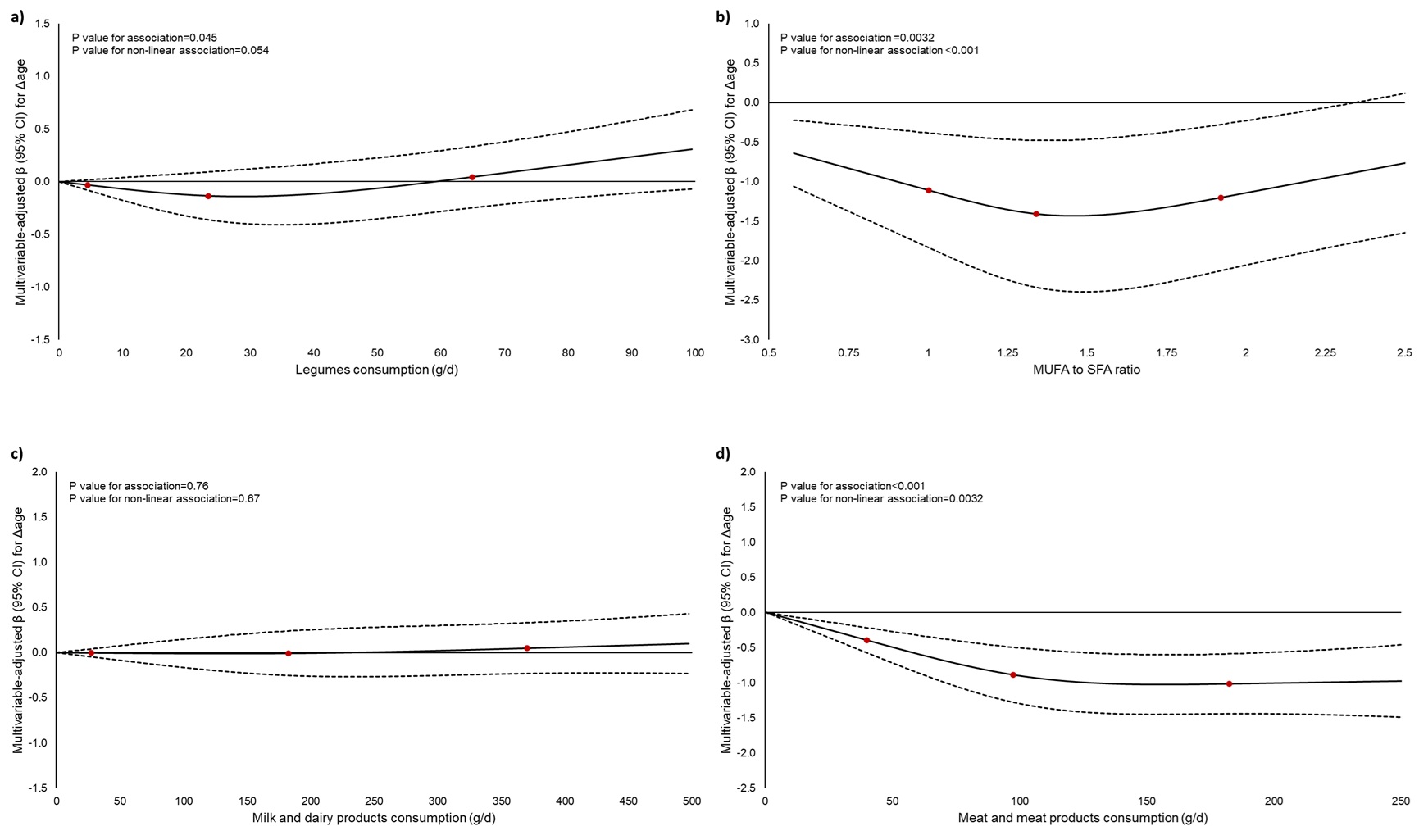
**

**Supplementary Appendix**

**Moli-sani Study Investigators**

The enrolment phase of the Moli-sani Study was conducted at the Research Laboratories of the Catholic University in Campobasso (Italy), the follow up of the Moli-sani cohort is being conducted at the Research Unit of Epidemiology and Prevention of the IRCCS Neuromed, Pozzilli, Italy.

**Steering Committee:** Licia Iacoviello*^#^ (Chairperson), Giovanni de Gaetano*, Maria Benedetta Donati*.

**Scientific Secretariat:** Chiara Cerletti* (Coordinator), Marialaura Bonaccio*, Americo Bonanni*, Simona Costanzo*°, Amalia De Curtis*, Augusto Di Castelnuovo*, Alessandro Gialluisi*^#^, Francesco Gianfagna°, Mariarosaria Persichillo*.

**Safety and Ethical Committee:** Jos Vermylen (Catholic University, Leuven, Belgio) (Chairperson), Renzo Pegoraro (Pontificia Accademia per la Vita, Roma, Italy), Antonio G. Spagnolo (Catholic University, Roma, Italy).

**External Event Adjudicating Committee**: Deodato Assanelli (Brescia, Italy), Livia Rago (Campobasso, Italy).

**Baseline and Follow-up Data Management:** Simona Costanzo*° (Coordinator), Sabatino Orlandi*, Teresa Panzera*.

**Data Analysis:** Augusto Di Castelnuovo* (Coordinator), Marialaura Bonaccio*, Francesca Bracone*, Simona Costanzo*°, Giuseppe Di Costanzo*, Simona Esposito*, Alessandro Gialluisi*^#^, Anwal Ghulam°, Francesco Gianfagna°, Martina Morelli*^†^, Maria Loreto Muñoz Venegas*^†^, Antonietta Pepe*, Emilia Ruggiero*^§^.

**Biobank, Molecular and Genetic Laboratory:** Amalia De Curtis* (Coordinator), Concetta Civitillo*^†^, Alisia Cretella*^†^, Sara Magnacca*.

**Recruitment Staff:** Mariarosaria Persichillo* (Coordinator), Francesca Bracone*, Giuseppe Di Costanzo*, Martina Morelli*^†^.

**Communication and Press Office:** Americo Bonanni*.

**Regional Institutions:** Direzione Generale per la Salute - Regione Molise; Azienda Sanitaria Regionale del Molise (ASReM, Italy); Agenzia Regionale per la Protezione Ambientale del Molise (ARPA Molise, Italy); Molise Dati Spa (Campobasso, Italy); Offices of vital statistics of the Molise region.

**Hospitals:** Presidi Ospedalieri ASReM: Ospedale A. Cardarelli – Campobasso, Ospedale F. Veneziale – Isernia, Ospedale San Timoteo - Termoli (CB), Ospedale Ss. Rosario - Venafro (IS), Ospedale Vietri – Larino (CB), Ospedale San Francesco Caracciolo - Agnone (IS); Casa di Cura Villa Maria - Campobasso; Responsible Research Hospital - Campobasso; IRCCS Neuromed - Pozzilli (IS).

*^*^Research Unit of Epidemiology and Prevention, IRCCS Neuromed, Pozzilli, Italy*

*^#^Department of Medicine and Surgery, LUM University “Giuseppe Degennaro”, Casamassima, Italy*

*^°^Department of Medicine and Surgery, University of Insubria, Varese, Italy*

^§^*Fellow of the Fondazione Umberto Veronesi, Italy*

*^†^Fondazione Veronesi – Piattaforma UMBERTO*

*Moli-sani Study Past Investigators are available at* <https://www.moli-sani.org/?page_id=173>
